# Harnessing non‑invasive vagal neuromodulation: HRV biofeedback and SSP for cardiovascular and autonomic regulation (Review)

**DOI:** 10.3892/mi.2025.236

**Published:** 2025-04-29

**Authors:** Asaf Gitler, Yoav Bar Yosef, Uri Kotzer, Ariel D. Levine

**Affiliations:** 1 Faculty of Social Welfare and Health Sciences, University of Haifa, Haifa 3498838, Israel; 2 Hematology Institute, Tel Aviv Sourasky Medical Center (Ichilov Hospital), Tel Aviv 6423906, Israel; 3 Kotzer Psychological Services, Ganei Tikva 5591166, Israel; 4 Department of Orthopedic Surgery, Bnai Zion Medical Center, Haifa 31048, Israel

**Keywords:** vagus nerve, biofeedback, stimulation, cardio-vascular, neuromodulation

## Abstract

The autonomic nervous system plays a central role in maintaining physiological homeostasis, with the vagus nerve as a crucial component. Heart rate variability (HRV) is a non-invasive biomarker of vagal tone and autonomic flexibility. A reduced HRV has been associated with cardiovascular diseases, hypertension, inflammation and mental health disorders. The present narrative review discusses two promising non-invasive interventions designed to enhance vagal activity: HRV biofeedback (HRV-B) and the safe and sound protocol (SSP). HRV-B utilizes paced breathing at an individual's resonance frequency combined with real-time HRV monitoring to strengthen baroreflex sensitivity, improve autonomic balance, reduce systemic inflammation and enhance emotional regulation. Extensive research supports the efficacy of HRV-B in improving cardiovascular outcomes, managing hypertension, reducing depressive and anxiety symptoms, and fostering resilience. By contrast, SSP is a relatively newer auditory-based intervention grounded in the polyvagal theory. It targets the ventral vagal complex through filtered music to promote social engagement, emotional regulation, and parasympathetic activation. Preliminary studies suggest SSP may benefit individuals with autism spectrum disorder, post-traumatic stress disorder and cardiovascular disease; however, large-scale controlled trials are needed to validate its clinical efficacy. By bridging cardiovascular, neurological and psychological domains, both HRV-B and SSP offer innovative non-invasive strategies for promoting health and resilience. The review highlights the mechanisms, clinical applications, and outcomes associated with each approach, while also discussing current limitations, such as individual variability, adherence challenges, and the need for further research. Future directions include integrating wearable HRV monitoring, AI-driven adaptive biofeedback, and expanding investigation across diverse populations to optimize clinical protocols. Together, HRV-B and SSP represent promising avenues for enhancing vagal neuromodulation in both preventive and therapeutic settings.

## 1. Introduction

The pursuit of health and well-being lies at the core of modern medicine. Beyond merely treating diseases, the medical field seeks to identify risk factors and protective mechanisms that prevent adverse health outcomes and foster a higher quality of life. Equally important is understanding the biological processes that sustain resilience and balance, shifting the focus from managing illness to actively enhancing the elements contributing to overall well-being. Globally, heart disease, cancer, stroke and chronic obstructive pulmonary disease remain the leading causes of mortality, imposing a tremendous burden on individuals and healthcare systems (1). Chronic conditions such as pain, anxiety and depression also significantly contribute to the global burden of disease due to their disabling effects (2). Addressing these challenges requires identifying shared risk factors and implementing cost-effective, evidence-based interventions to improve outcomes, reduce disability, and promote longevity.

The COVID-19 pandemic has underscored the importance of autonomic regulation, as SARS-CoV-2 infection is associated with dysautonomia, inflammatory cytokine storms and long-term autonomic dysfunction (long COVID). Reduced heart rate variability (HRV) has been observed in patients recovering from COVID-19, indicating impaired vagal function (3,4). HRV biofeedback intervention (HRV-B) and the safe and sound protocol (SSP) could potentially serve as non-invasive rehabilitative strategies to restore autonomic balance, mitigate post-viral fatigue and improve cardiovascular function. Preliminary studies suggest that vagus nerve stimulation (VNS) may counteract COVID-19-induced inflammation by modulating the cholinergic anti-inflammatory pathway, reducing excessive cytokine responses. Given the ability of HRV-B and SSP to enhance vagal tone, future research should investigate their role in post-COVID recovery.

Seamless communication between vital systems, orchestrated by the autonomic nervous system (ANS), is fundamental to a healthy organism. The ANS, comprising the sympathetic nervous system and parasympathetic nervous system (PNS), maintains homeostasis by regulating responses to internal and external stimuli (5,6). This dynamic system synchronizes physiological functions and serves as a crucial interface between neurophysiological processes and peripheral markers of disease.

Central to the PNS is the vagus nerve, aptly termed the ‘wandering nerve’ for its expansive reach. Composed of 80% afferent and 20% efferent fibers, it transmits signals from the organs of the body to the brain and regulates organ functions (7,8). Originating from the brainstem, the vagus nerve extends through the neck and thorax, innervating key visceral organs such as the heart, lungs, liver, and colon. Descending fibers from the nucleus ambiguous and dorsal vagal nucleus synapse at cardiac plexuses, influencing cardiac structures, such as the sinoatrial node, atrioventricular node and coronary arteries (9).

The vagus nerve plays a pivotal neuro-immunological role, facilitating communication between the nervous and immune systems. Immune cells express neurotransmitter receptors, while the nervous system innervates lymphoid tissues, enabling the vagus nerve to modulate inflammation via two critical pathways. The hypothalamic-pituitary-adrenal (HPA) axis mediates cortisol release and systemic anti-inflammatory effects. By contrast, the cholinergic anti-inflammatory pathway involves splenic T-cells releasing acetylcholine to suppress inflammatory cytokine production (10-12).

This dual role highlights the potential of the vagus nerve as a therapeutic target. Its ability to regulate inflammation and promote physiological coherence offers profound implications for improving health and reducing global disease burden.

### The vagal cardio-respiratory role

The vagus nerve regulates the dynamic interplay between cardiac and respiratory functions. Respiratory sinus arrhythmia (RSA), a physiological phenomenon where the heart rate accelerates during inhalation and decelerates during exhalation, exemplifies this interaction. This variation is driven by vagal modulation, showcasing the vital role of the PNS in maintaining cardiovascular and respiratory balance (13,14).

HRV, which measures the intervals between heartbeats, captures the dynamic balance between sympathetic and parasympathetic inputs to the heart. RSA constitutes a key component of HRV, reflecting how the vagus nerve fine-tunes cardiac activity to align with respiratory patterns. This synchronization is a marker of physiological coherence and a critical predictor of survival in conditions, such as coronary heart disease (CHD) (14).

Vagal activation during exhalation decreases the respiratory rate and enhances the parasympathetic tone. This process promotes efficient gas exchange, demonstrating the broader role of the vagus nerve in fostering resilience and adaptability within the cardio-respiratory system. RSA and HRV are invaluable tools for assessing autonomic health and designing interventions to optimize overall well-being (15).

### Association between vagal activity and the brain

The connection between the brain and the heart is a finely tuned interplay of cognitive, emotional and autonomic processes, with the vagus nerve playing a central role in this sophisticated communication network. The neurovisceral integration model provides a framework for understanding this association, emphasizing vagal-mediated HRV (vmHRV) as a crucial link between autonomic regulation and higher-order brain functions. A higher vmHRV indicates an enhanced ability of the brain to regulate emotions, adapt cognitively and manage stress, underscoring the intertwined nature of mental and physical health (16,17).

Neuroimaging studies and meta-analyses have substantiated this model by revealing correlations between HRV metrics and activity in critical brain regions, including the amygdala, cingulate cortex and prefrontal cortex. These areas are essential for evaluating safety and threat, regulating emotions and executing cognitive tasks, such as memory and decision-making. It has been further demonstrated that vmHRV is associated with the structural and functional integrity of these brain regions, highlighting the multi-level integration of brain-heart dynamics (18,19).

HRV is more than a mere indicator of cardiac activity; it provides insight into the capacity of the brain to regulate physiological states effectively. Time-domain measures, such as the root mean square of successive differences (RMSSD) and frequency-domain measures, such as high-frequency (HF) power capture the influence of the vagus nerve on the PNS. These measures reflect immediate autonomic responses and long-term biological rhythms, providing a comprehensive view of autonomic function (20-27).

The parasympathetic role of the vagus nerve also extends to counteracting sympathetic overactivity. The vagus enhances coronary blood flow and mitigates hypoxia by inducing vasodilation and releasing vasoactive intestinal peptides. This function is particularly significant in reducing the risks associated with CHD, stroke and cancer, as hypoxia contributes to tumor progression and poor prognosis in cancer patients (23,24).

Higher HRV levels, mediated by the vagus nerve, signify robust autonomic balance, emotional regulation and cognitive function. These attributes enhance resilience to stress and disease, positioning vmHRV as a vital marker of overall well-being by bridging the domains of neural regulation and cardiovascular health.

### HRV: The heart's window into the vagus nerve

HRV is a non-invasive marker that provides profound insight into vagal nerve activity, often termed vagal tone. HRV reflects the interplay between autonomic inputs and cardiac function by analyzing the fluctuations between successive heartbeats. This variability is strongly associated with the ability of the body to adapt to metabolic demands and external challenges, rendering it an essential indicator of physiological resilience (25-29).

HRV is commonly measured using electrocardiography (ECG) or photoplethysmography, with sensors placed on the earlobe, chest, or finger. Its strong correlation with vagal nerve activity (r=0.88) validates its reliability as a proxy for autonomic function (25). Specific metrics, such as RMSSD and HF power are particularly indicative of vmHRV, which reflects parasympathetic cardiac control (25,27,28).

HRV analysis can be categorized into time and frequency domains. Time-domain measures, such as standard deviation of normal-to-normal intervals (SDNN) and RMSSD, evaluate interval variability between successive heartbeats, providing valuable insight into autonomic regulation. While 5-min recordings are standard for HRV analysis, shorter HRV recordings, such as ultra-short (10-30 sec) and short-term (1-5 min) assessments, have gained popularity due to their practicality in clinical settings. They offer rapid assessments, making HRV measurement accessible in routine medical evaluations. However, these shorter measurements may not fully capture long-term autonomic trends or provide the same prognostic power as 24-h HRV recordings. Research suggests that RMSSD and HF-HRV from short-term recordings are reliable indicators of parasympathetic activity. Still, metrics such as low-frequency (LF)-HRV and SDNN require more prolonged monitoring for better accuracy. Shorter 10-sec recordings have gained traction in clinical settings due to their practicality and strong correlation with longer recordings, particularly for RMSSD (15,30).

The frequency-domain analysis divides heart rate oscillations into specific frequency bands, each representing physiological processes. Ultra-LF (ULF) bands capture slow biological rhythms, such as circadian and metabolism, primarily observed in 24-h recordings. The very-LF (VLF) band reflects influences, such as physical activity, temperature regulation and autonomic control, with rhythms lasting for 25 to 300 sec. LF oscillations are linked to baroreflex activity and involve both sympathetic and parasympathetic influences, while HF bands are entirely under vagal control, correlating strongly with RSA (15,25,29).

Total HRV power, which includes ULF, VLF, LF and HF components, provides a holistic view of autonomic activity over a period of 24 h, although shorter-term components are commonly employed in clinical practice.

HRV is a powerful predictor of health and disease outcomes, demonstrating utility across various clinical conditions, including cardiovascular disease, post-traumatic stress disorder (PTSD) and neurodevelopmental disorders. HRV-B has been extensively studied in autonomic regulation, demonstrating significant benefits in managing hypertension, depression and stress-related disorders. By contrast, SSP, although still emerging, has exhibited preliminary promise in autism spectrum disorder (ASD) and PTSD. An elevated HRV is associated with enhanced resilience and recovery from stress, reflecting efficient hormonal, inflammatory and cardiac regulation, and by contrast, a reduced HRV is linked to an increased risks of heart failure, cancer and all-cause mortality, underscoring its value as a prognostic marker (31-34). Additionally, HF-HRV and related HRV metrics have gained recognition as biomarkers for mental stress and psychopathology, highlighting the intersection between psychological and physiological health (35-37). A summary of key disorders, associated symptoms, biomarkers and study references is provided in Table I.

Interrelationships between time-domain and frequency-domain metrics further enhance the utility of HRV. For example, SDNN is closely linked to LF power, while RMSSD is strongly associated with HF power, particularly during sleep (38). RMSSD remains unaffected by respiration, strengthening its reliability as a vagal tone marker, even as respiratory changes influence HF measures (39-41).

This multifaceted approach to HRV underscores its broad applicability in diagnosing and managing autonomic dysfunctions, promoting health and understanding the complex interactions between the heart and vagus nerve. HRV is invaluable for advancing physical and psychological resilience and improving disease outcomes.

### Role of HRV in health and diseases

HRV serves as a vital marker of overall health, with significant implications for diagnosing and predicting the progression of various diseases. High HRV, measured through parameters, such as very-low-frequency power (P<0.0001), low-frequency power (P<0.0001), high-frequency power (P=0.0014), total power (P<0.0001) and the standard deviation of total RR intervals (P=0.0019), has consistently been found to be associated with a reduced all-cause mortality (42).

For instance, individuals with an SDNN <70 msec following myocardial infarction face an almost 4-fold greater risk of mortality within 3 years compared to those with higher SDNN values, as previously demonstrated in a meta-analysis (43). HRV also has predictive power in patients with cancer, with SDNN values being significantly lower in affected individuals (22 msec) than in their healthy counterparts (50 msec). Elevated vagal nerve activity, indicated by higher SDNN levels, is strongly associated with extended survival periods in these populations (44,45).

Chronic diseases and adverse health outcomes, such as hypertension (46,47), systemic inflammation (48,49) and diabetes (50), are frequently associated with a reduced HRV in both time and frequency domains. While the broad spectrum of conditions linked to HRV highlights its importance, it also underscores the challenge of using HRV to diagnose illnesses. This lack of specificity likely stems from the complex interplay of biological mediators, particularly inflammation (51).

Inflammation, a common factor in numerous diseases, including cardiovascular conditions and cancer, is strongly linked to HRV metrics. Previous studies have revealed a consistent inverse associations between HRV and inflammatory markers across various age groups (49,52-55). For example, a prospective study demonstrated that baseline HF-HRV inversely correlated with C-reactive protein (CRP) levels, an inflammatory marker, and predicted CRP levels up to 4 years later (56). These findings suggest the utility of HRV as a dynamic indicator of inflammation.

HRV has proven to be a reliable predictor of outcomes in surgical settings. Patients with a lower pre-operative HRV tend to have prolonged hospital stays (57), poorer pain control (58) and slower recovery rates. Moreover, pre-operative HRV levels are closely tied to postoperative CRP trajectories, with lower HRV linked to higher CRP levels and delayed inflammation resolution during recovery. The pace of inflammatory decline between post-operative days is also associated with the duration of hospitalization, emphasizing the role of HRV in predicting surgical recovery (59).

As a reflection of vagus nerve activity, vmHRV captures a wide array of psychobiological and neurophysiological processes. Its predictive power extends to significant global health burdens, providing insight into disease onset and progression. Established norms for short-term and ultra-short HRV measurements provide valuable benchmarks for clinical and research applications across diverse populations and conditions (60-63).

## 2. History and clinical application of vagus nerve stimulation

The therapeutic potential of VNS has substantially evolved since its discovery in the late 19th century when James Corning first explored its anti-seizure effects in epileptic patients (64). Over time, various non-pharmacological methods have been developed to harness the therapeutic benefits of the vagus nerve, addressing conditions ranging from chronic headaches (65,66) and inflammation (67) to depression and atrial fibrillation (68).

VNS techniques are categorized into two main approaches: Invasive VNS (iVNS) and non-invasive VNS (nVNS). International consensus recognizes four primary VNS modalities: Cervically implanted (iVNS), transcutaneous cervical VNS (tcVNS), transcutaneous auricular VNS (taVNS) and percutaneous auricular VNS (paVNS) (69). iVNS involves surgically implanting a pulse generator under the skin of the upper chest, and connecting it to the left vagal nerve via electrodes (70-72). Conversely, nVNS stimulates vagal nerve pathways accessible through the skin, typically targeting the external ear (taVNS) or the neck (tcVNS) (73-75).

In 1997, iVNS received FDA approval for the treatment of medically refractory partial epilepsy, followed by its use in severe, recurrent unipolar and bipolar depression in 2005 (76,77). In Europe, iVNS achieved the Conformité Européenne (CE) marking for epilepsy and treatment-resistant depression (77,78). However, the surgical risks and side-effects associated with iVNS prompted the development of non-invasive alternatives.

nVNS provides comparable benefits, while improving safety and accessibility (79,80). Introduced clinically in 1997, nVNS mimics the physiological effects of iVNS, although with improved tolerability and fewer adverse effects (81). The FDA has approved tcVNS for the treatment of migraines (82) and cluster headaches (74). Over the years, VNS has been used in the treatment of hundreds of thousands of patients with various neurological disorders, generating extensive clinical data for exploring new applications.

Stroke rehabilitation represents a promising area for VNS. Randomized controlled trials have indicated that combining VNS, both invasive and non-invasive, with rehabilitation therapy significantly enhances upper limb recovery post-stroke (72,83,84). Additionally, VNS has shown potential in managing chronic pain, tinnitus, epilepsy, and depression (85-90). Notably, the VNS-REHAB study, published in *The Lancet*, highlighted VNS as an innovative treatment for limb paralysis caused by ischemic stroke, solidifying its role in neurorehabilitation (72).

Non-invasive VNS stands out due to its ease of implementation and favorable safety profile. By eliminating surgical risks, such as infections, peritracheal hematomas, vocal cord damage and dyspnea, nVNS has become a patient-friendly alternative (79). Techniques, such as auricular branch stimulation have proven effective in treating atrial fibrillation, reducing sinus rates, slowing atrioventricular conduction and shortening atrial fibrillation episodes (91,92). Similarly, neck-based nVNS has demonstrated anti-inflammatory properties, broadening its therapeutic utility (93). Furthermore, nVNS is simple, cost-effective, and safe with ECG and HRV monitoring, making it accessible to many patients.

VNS continues to evolve as a versatile therapeutic tool. Its non-invasive modalities enhance accessibility and safety, paving the way for broader clinical applications in managing neurological and systemic conditions. As ongoing research refines these techniques, VNS holds immense promise for advancing patient care and outcomes.

## 3. Vagus nerve stimulation via heart rate variability biofeedback

HRV-B is an innovative, non-invasive and evidence-based technique designed to enhance vagal nerve activity by combining slow-paced breathing with real-time HRV feedback. The practice involves regulating breathing to approximately six cycles per minute, significantly lower than the standard respiratory rate of 12 to 20 breaths per minute (94). By synchronizing this controlled breathing pattern with HRV feedback, individuals can effectively boost vagal tone, improving overall autonomic regulation. HRV-B is simple to implement, cost-effective, and carries minimal risk, rendering it an accessible tool for various health interventions. Established protocols further support its practical application (95,96).

This method emphasizes the importance of breathing education, focusing on techniques that lower respiratory rates, relax thoracic muscles, and promote diaphragmatic breathing. Tension in the abdomen, pelvis, or lower back often restricts diaphragmatic movement, leading to reliance on chest and shoulder muscles for respiration. Such inefficient breathing patterns can contribute to hyperventilation, characterized by excessive breathing and reduced end-tidal CO2 levels. Hyperventilation, in turn, is strongly associated with psychological stress and various somatic and behavioral symptoms (97-99). By contrast, slow-paced breathing promotes relaxation and resilience, offering preventive and adjunctive benefits for numerous conditions with minimal adverse effects (100).

HRV-B influences autonomic regulation primarily through baroreflex engagement and the cholinergic anti-inflammatory pathway. At the cellular level, HRV-B enhances the activity of vagal afferents, inhibiting pro-inflammatory cytokines, such as TNF-α, IL-6 and IL-1β through the release of acetylcholine from splenic T-cells. This mechanism directly attenuates systemic inflammation and promotes cardiovascular resilience (15).

HRV-B modulates the activity of the HPA axis, reducing cortisol secretion and enhancing parasympathetic outflow. The resulting physiological state supports neuroplasticity by strengthening prefrontal cortex connectivity with autonomic centers in the brainstem. SSP, although still under investigation, is considered to engage vagal activity through auditory-motor circuits. It likely modulates neuroplasticity within the nucleus ambiguous and dorsal vagal complex, enhancing parasympathetic tone. Preliminary evidence suggests that SSP may influence oxytocin release, contributing to social bonding and stress resilience; however, further studies are required to confirm these cellular-level effects (101-105). HRV-B primarily operates through baroreflex engagement, stabilizing autonomic rhythms and enhancing vagal tone (15).

This results in improved cardiac efficiency, reduced sympathetic overactivation and lower systemic inflammation. On the other hand, SSP engages the ventral vagal complex via auditory-motor pathways, facilitating neuroplasticity and enhancing emotional regulation. Recent research suggests that SSP may function by modulating the influence of the prefrontal cortex on autonomic outflow, promoting a shift toward parasympathetic dominance (101-105).

HRV-B uses audiovisual biofeedback tools to present real-time data on physiological parameters, such as heart rate, muscle activity, brain activity and skin conductance. This feedback empowers individuals to consciously regulate these processes, enhancing psychological and physiological well-being (28). The technique explicitly trains individuals to adopt optimal breathing patterns, significantly increasing vmHRV. By doing so, HRV-B activates autonomic and respiratory regulatory mechanisms, fostering physiological coherence. HRV-B training aims to achieve a steady breathing rate of around six cycles per minute. Reviewing key quantitative metrics across studies to provide a more precise assessment of HRV-B and SSP effectiveness is essential (101-105).

For HRV-B, commonly reported parameters include RMSSD, HF-HRV and SDNN, which indicate vagal activity. SSP studies often measure changes in resting HF-HRV and LF/HF ratios to assess autonomic modulation. The results from major studies are compared in Table II. These results highlight the measurable effects of HRV-B and SSP on autonomic balance.

Although individual variations may exist (106), this synchronization of heart rate oscillations with respiratory patterns leads to a state known as cardiac, respiratory and blood pressure coherence, or HRV coherence (106-108). During this state, frequency-domain metrics often reflect a dominance of the low-frequency (LF) band centered around 0.1 Hz, with decreases in HF and VLF bands and mean heart rate. HRV-B sessions provide visual and auditory representations of heart rate and respiration, helping individuals maintain and amplify LF-HRV power (109).

HRV-B strengthens vagal tone and promotes broader autonomic resilience by fostering physiological coherence, rendering it a powerful tool for managing stress, enhancing mental health and improving overall well-being. Its simplicity, accessibility and efficacy contribute to its value in clinical practice and personal health management. The intervention primarily operates through baroreflex engagement, vagal afferent activation, and modulation of the cholinergic anti-inflammatory pathway, leading to the suppression of pro-inflammatory cytokines, such as TNF-α, IL-6 and IL-1β, which play a crucial role in systemic inflammation and autonomic dysfunction. By contrast, SSP is hypothesized to engage vagal pathways via auditory-motor circuits, potentially enhancing neuroplasticity within autonomic regulatory centers and thereby supporting improved emotional and physiological regulation. The underlying mechanisms, physiological effects, and clinical benefits of HRV-B and SSP are summarized in Table III.

### Resonance frequency breathing and its clinical implications

Resonance-frequency breathing is an individualized approach grounded in the unique resonance properties of the cardiovascular system, particularly the baroreflex, a vagally mediated feedback mechanism that regulates blood pressure through heart rate modulation (110). The delay caused by inertia in the blood supply determines this resonance frequency. Breathing at one's specific resonance rate, typically between 4.5 and 6.5 breaths per minute, systematically activates the baroreflex, synchronizing heart rate and respiration. This synchronization improves gas exchange efficiency and enhances oxygen saturation (108,111-114).

Through consistent practice, particularly when combined with biofeedback, individuals can align three physiological oscillators, baroreflex, blood pressure and respiration, at this resonance frequency. This alignment shifts the peak frequency of HRV from the HF range (~0.2 Hz) to the LF range (0.04-0.15 Hz), typically corresponding to six breathing cycles per minute, with each inhalation and exhalation lasting ~10 sec. Biofeedback training at the resonance frequency produces immediate, large-scale increases in baroreflex gain, a benefit that persists over time with continued practice, even without ongoing biofeedback (95).

The technique has demonstrated its potential to modulate the cholinergic anti-inflammatory pathway (115,116). A pilot study involving 10 participants revealed that HRV-B performed at the resonance frequency significantly reduced CRP levels, a marker of systemic inflammation, compared to HRV-B performed at usual breathing rates (117). Similarly, randomized controlled trials have highlighted the impact of slow-paced breathing (SPB) combined with HRV biofeedback on inflammatory markers. A previous study divided 55 participants into two groups: An intervention group practicing SPB with HRV biofeedback (SPB-HRV-BF) and a control group using HRV-sham biofeedback (118). Over a period of 4 weeks, SPB-HRV-BF significantly reduced the levels of TNF-α [F(1, 53)=4.396, P≤0.05, η2=0.077], while increasing HRV metrics, such as SDNN, total power and LF during resting conditions (118). These findings underscore the therapeutic potential of SPB-HRV-BF in managing inflammation.

Numerous meta-analyses and systematic reviews have provided evidence to indicate that the clinical significance of HRV-B extends to various disorders. These have studies consistently demonstrated that HRV-B significantly reduces self-reported stress and anxiety levels, alleviates depressive symptoms, and enhances emotional and physical functioning (119-121). A recent systematic review further highlighted the benefits of HRV-B in addressing hypertension, improving cardiovascular outcomes, reducing inflammation and alleviating pain (122).

The role of HRV-B in mental health, particularly its efficacy in treating anxiety and depression, has been well-documented. HRV-B provides a robust, non-invasive intervention with significant therapeutic potential by leveraging vagal nerve activity and promoting resonance frequency breathing. Further exploration of its mechanisms will deepen the current understanding of how vagal nerve modulation contributes to health and resilience, paving the way for broader applications in clinical practice and disease prevention (28).

## 4. Vagal tone, HRV and HRV biofeedback in heart diseases

HRV has emerged as a critical indicator of cardiovascular health, particularly in predicting and managing CHD. A reduced HRV is consistently linked with an elevated risk of cardiac events. A prospective study involving 2,501 participants without pre-existing heart disease revealed that a decrease in the SDNN independently predicted a 47% higher risk of future cardiac events (42). Supporting these findings, a meta-analysis of 21 studies revealed that patients with SDNN values <70 msec following a myocardial infarction faced an almost 4-fold greater risk of mortality compared to those with higher SDNN values (43). Further evidence from a review of nine studies demonstrated that patients who experienced ST-elevation myocardial infarction and had low HRV in the time domain or a high LF/HF ratio were at a significantly higher risk of all-cause mortality and major adverse cardiovascular events (123).

The physiological mechanisms linking reduced HRV to cardiovascular diseases involve oxidative stress, heightened sympathetic nervous system activity, and inflammation. Oxidative stress contributes to oxidized low-density lipoprotein (LDL) cholesterol formation, while inflammation attracts macrophages to developing cardiac plaques. These plaques engulf oxidized LDL, worsening coronary blockages (124,125). Excessive sympathetic activity exacerbates the risk of a myocardial infarction by increasing oxygen demand, causing coronary vasoconstriction and aggravating coronary lesions. Conversely, as higher HRV indicates, vagal nerve activity mitigates these risks by reducing oxidative stress, regulating inflammation and curbing sympathetic overactivity (12,51,126).

HRV-B provides promising therapeutic benefits for individuals with myocardial infarction or CHD by directly improving HRV and modulating autonomic responses. In a previous study on 54 with coronary artery disease (CAD), those randomized to HRV-B therapy exhibited significant improvements in HRV and reduced hostility, independent predictors of better outcomes post-MI (127,128). Another randomized controlled trial demonstrated that HRV-B significantly increased LF-HRV and SDNN levels in patients with CHD (129). Similarly, another randomized controlled trial involving 46 patients post-myocardial infarction found that HRV-B enhanced HRV metrics (SDNN and HF-HRV) and improved self-efficacy, while no such benefits were observed in the control group (130).

Further evidence was derived from a year-long study of 210 Chinese patients with CAD, half of whom underwent HRV-B therapy; the HRV-B group exhibited a significantly increased LF-HRV and experienced reduced hospital readmissions at the 1-year follow-up (131). Another randomized controlled trial that combined HRV-B with cognitive-behavioral stress management training demonstrated that improvements in psychological adjustment were closely linked to increases in the HF index of vagal HR modulation, but only in the HRV-B group (132).

These findings establish HRV-B as an effective intervention for enhancing HRV, improving psychological well-being and reducing hospital readmissions in patients with myocardial infarction, CAD, or CHD. By addressing underlying mechanisms, such as inflammation and oxidative stress, HRV-B provides a valuable tool for the management of and recovery from cardiovascular disease.

## 5. Vagal tone, HRV and HRV biofeedback in hypertension

Several large-scale epidemiological studies have demonstrated that hypertension has also been closely linked to a reduced HRV. For instance, the Atherosclerosis Risk in Communities (ARIC) study followed 7,099 initially normotensive individuals and found that reductions in SDNN and the RMSSD were strong predictors of hypertension development over a period of 9 years (133). These findings underscore the role of diminished HRV as a significant risk factor for elevated blood pressure.

The vagus nerve plays a central role in regulating blood pressure through the baroreflex, a negative feedback mechanism critical for maintaining hemodynamic stability. Mechanoreceptors in large vessels, such as the aorta, detect changes in arterial pressure and activate the vagus nerve, reducing the heart rate via brainstem pathways and lowering cardiac output and blood pressure. Additionally, while vasoconstriction contributes to hypertension, the vagus nerve counteracts this through its vasodilatory effects, further aiding in blood pressure regulation.

The therapeutic potential of HRV-B in managing hypertension is well-supported by randomized controlled trials. In a notable 3-month study of 43 pre-hypertensive participants, those randomized to HRV-B demonstrated significant increases in HRV and reductions in both systolic blood pressure and diastolic blood pressure, unlike participants in the deep breathing or no-intervention groups (134). That study also highlighted the importance of biofeedback in reinforcing and self-monitoring physiological changes, showing its added value over simple-paced breathing (134).

Another randomized controlled trial involving 65 hypertensive patients found that HRV-B significantly reduced systolic blood pressure compared to autogenic relaxation, which was used as a control intervention (132). Similarly, a smaller study on 22 participants explored the effects of R-wave-to-pulse interval biofeedback, a method conceptually similar to HRV-B, over a period of 2 weeks (135). Among the 12 participants with high blood pressure, diastolic levels significantly decreased during the intervention. Notably, participants with low blood pressure experienced increases in diastolic levels, although the absence of a control group in that study complicates the interpretation of these results (135).

These findings provide compelling evidence of the effectiveness of HRV-B in regulating blood pressure. By enhancing vagal tone and improving baroreflex sensitivity, HRV-B serves as a promising non-pharmacological strategy for managing hypertension. Its ability to address the underlying autonomic dysfunction associated with elevated blood pressure highlights its potential to improve cardiovascular health and overall well-being.

## 6. Vagus nerve stimulation via the safe and sound protocol

The SSP, founded on the principles of the polyvagal theory, represents a groundbreaking, non-invasive approach to vagus nerve stimulation. This protocol capitalizes on the central role of the vagus nerve in maintaining health by focusing on three critical aspects. First, it highlights the connection between the ventral vagal complex and symptom relief, demonstrating how stimulating specific afferent pathways can significantly enhance mental and physical health (101). Second, SSP engages afferent pathways linked to brainstem regions that regulate somatomotor and visceromotor responses, thereby optimizing the autonomic nervous system's regulatory functions (102). Lastly, the protocol acknowledges the profound impact of chronic stress and trauma in disrupting the autonomic balance. These disruptions often hinder the ability of the ventral vagal complex to manage threat responses and maintain the homeostasis essential for growth, recovery and overall health (103).

Advances in understanding the anatomy and physiology of the vagus nerve have driven the evolution of clinical strategies such as neuromodulation. These strategies emphasize exercises to strengthen autonomic function and reduce dependence on long-term interventions (104). SSP employs specially filtered music to stimulate the vagus nerve via the auditory system, fostering a profound sense of physiological safety. This stimulation mitigates defensive responses, such as anxiety and stress, while improving emotional regulation and social engagement. By targeting the middle ear muscles essential for processing human speech, SSP enhances individuals' sensitivity to social cues, improves communication, and fosters a sense of security in social interactions (103). Additionally, the protocol increases parasympathetic activity, stabilizing the autonomic nervous system. Studies have confirmed that SSP significantly enhances HRV, a key marker of autonomic regulation, thus promoting stress resilience and overall well-being (103,105,136).

The SSP is a relatively new intervention based on the principles of the polyvagal theory. SSP aims to influence autonomic function by stimulating auditory pathways linked to vagal regulation. While preliminary studies have reported improvements in autonomic markers and emotional regulation, the existing research is limited to small-scale studies, pilot trials and case reports, often without control groups. The effects of SSP on HRV and vagal tone remain an area of ongoing investigation. Thus, further, randomized controlled trials are warranted to determine its efficacy and applicability in clinical settings. Although some findings suggest that SSP may enhance social engagement and reduce stress responses, these results need to be interpreted with caution until more extensive and rigorous studies confirm its benefits.

In children with ASD, SSP has led to significant improvements in sensory processing, particularly in auditory function, with substantial results observed after only 5 h of exposure to filtered music (105,136). Beyond autism, SSP has gained recognition for its potential in cardiovascular disease rehabilitation. Research consistently associates a low HRV with increased mortality rates in cardiovascular conditions, while chronic stress exacerbates the risk of developing cardiovascular disease by promoting central adiposity, metabolic dysregulation and inflammation (137,138). The stress-buffering hypothesis underscores the critical role of social support in mitigating the physiological impacts of stress, with social engagement identified as a key prognostic factor for cardiovascular health (139,140). By enhancing parasympathetic activity and HRV, SSP is a valuable intervention for counteracting stress-related effects in CVD patients. Its ability to foster social engagement and reduce defensive responses aligns with the stress-buffering hypothesis, suggesting a role of SSP as an integral component of cardiovascular rehabilitation programs. By promoting emotional and physiological regulation, SSP helps patients better manage stress, improving cardiovascular and general health outcomes (138-140).

## 7. Special considerations: PTSD and heart diseases

The intricate association between PTSD and cardiovascular conditions is being increasingly evidence in research, revealing a dual dynamic where PTSD contributes to the onset of heart diseases and exacerbates their progression (141,142). Studies on veteran populations have demonstrated that PTSD significantly increases the risk of heart failure. For example, a previous study found a 47% greater risk of developing heart failure among veterans with PTSD, independent of traditional risk factors, such as diabetes and hypertension (143).

Notably, cardiac conditions themselves can precipitate PTSD, a phenomenon termed cardiac disease-induced PTSD (CDI-PTSD). Acute cardiovascular events such as myocardial infarction or cardiac arrest often function as triggers, given their life-threatening nature. CDI-PTSD differs from conventional PTSD by its distinct focus on an intense fear of recurrence and intrusive, future-oriented thoughts. For example, studies estimate that up to 25% of cardiac arrest survivors develop PTSD (144). Similarly, takotsubo syndrome (TTS), also known as ‘broken heart syndrome’, is frequently linked to emotional trauma. Although TTS typically resolves without causing long-term physical damage, its psychological effects can persist, leading to PTSD-like symptoms in some individuals (145).

HRV-B provides a promising therapeutic approach for managing both PTSD and cardiac conditions. This intervention enhances the vagal tone and restores autonomic nervous system regulation, addressing the dysregulation often seen in PTSD. HRV-B has shown particular efficacy in reducing PTSD symptoms, particularly in military populations (146). Simultaneously, HRV-B improves HRV and lowers the risk of recurrent cardiac events in individuals with cardiovascular disease (147).

The dual effectiveness of vagal neuromodulation techniques such as HRV-B in treating PTSD and cardiac conditions highlights its transformative potential. HRV-B provides an integrated approach that can significantly improve patient outcomes by addressing the psychological and physiological dimensions of managing these interrelated conditions.

## 8. Conclusion

The present article discussed two innovative, non-invasive approaches, namely HRV-B and SSP, focusing on their ability to enhance vagal tone and support autonomic regulation. The reviewed body of research underscores the importance of HRV as a prognostic indicator across diverse medical conditions, including myocardial infarction and cancer. While the broad applicability of HRV reduces its specificity for diagnosing particular illnesses, it remains a valuable tool for predicting and improving health outcomes. Employing feasible cardiovascular measurement and stimulation techniques based on HRV presents promising opportunities for advancing physical and mental health. These interventions also lay the groundwork for future research in vagal nerve stimulation and its clinical applications.

The present narrative review consolidates current knowledge and paves the way for future interdisciplinary exploration. By juxtaposing HRV-B and SSP within a cohesive model, it highlights novel avenues for enhancing resilience and autonomic regulation across various health conditions.

HRV-B is a well-established intervention for optimizing autonomic regulation, whereas SSP, although promising, requires further research to confirm its efficacy. Leveraging the crucial regulatory functions of the vagus nerve provides innovative pathways for addressing cardiovascular and mental health conditions. Beyond treatment, they hold immense potential for disease prevention and recovery, reinforcing their role in holistic healthcare approaches. Future research is required refine these techniques, broaden their clinical applications and uncover their full therapeutic potential.

## Figures and Tables

**Table I:** HRV-B and SSP applications in clinical disorders.

Pathology/disorder	Symptoms	Biomarkers	Patient population	Authors, year of publication	(Refs.)
Hypertension	Elevated blood pressure	SDNN, RMSSD, blood pressure levels	Hypertensive patients	Nolan *et al*, 2010	(132)
				Lin *et al*, 2012	(134)
				Rau *et al*, 2003	(135)
PTSD	Hypervigilance, intrusive memories, emotional dysregulation, sleep disturbances, heightened arousal	HF, RMSSD	PTSD among military service members (in previously published clinical trials)	Kenemore *et al*, 2024	(146)
Depression	Low energy, anhedonia, emotional dysregulation	SDNN, HF	Adults with MDD (in previously published clinical trials)	Lehrer and Gevirtz, 2014	(109)
Autism (SSP)	Sensory dysregulation, social and language difficulties, digestive issues, and selective eating	No biomarkers measured; outcomes based on subjective reports	Children, adolescents, and adults with ASD	Kishimoto *et al*, 2017;	(105)
				Heilman *et al*, 2023	(136)
Post-COVID dysautonomia	Fatigue, breathlessness palpitations, dizziness, pain, and cognitive fog	RMSSD	Long-COVID patients	Corrado *et al*, 2024	(4)

The table presents an overview of the clinical applications of HRV-B and the SSP across various disorders. It outlines the symptoms associated with each condition, relevant biomarkers used in research and clinical assessment, the patient populations most studied, and key references supporting the use of HRV-B and SSP in these populations. HRV, heart rate variability; HRV-B, heart rate variability biofeedback; SSP, safe and sound protocol; SDNN, standard deviation of normal-to-normal intervals; LF, low frequency; HF, high frequency; RMSSD, root mean square of successive differences; RSA, respiratory sinus arrhythmia.

**Table II:** Summary of HRV-B and SSP studies on HRV metrics.

Authors, year of publication	Intervention	HRV metric	Outcome	(Refs.)
Herhaus *et al*, 2023	HRV-B	SDNN, LF	Reduced pro-inflammatory cytokines (TNF-α); increased SDNN and LF-HRV after 4-week intervention	(118)
Lin *et al*, 2012	HRV-B	SDNN	Reduced systolic and diastolic blood	(127)
Lin *et al*, 2015		RMSSD, LF	pressure; increased HRV in hypertensive individuals	(134)
Kishimoto *et al*, 2023	SSP	HRV not directly measured	Reduced repetitive behaviors and improved sensory processing	(136)
Kawai *et al*, 2023	SSP	HRV not directly measured	Improvement in SRS-2 (Social Awareness); correlated with anxiety and depression measures	(103)

The table presents a summary of key studies evaluating the effects of HRV-B and SSP on HRV metrics. It includes the specific HRV parameters measured in each study and the observed changes following the intervention. HRV metrics, such as RMSSD and HF-HRV are commonly used indicators of parasympathetic (vagal) activity. At the same time, SDNN reflects overall autonomic balance. The LF/HF ratio indicates sympathetic-parasympathetic balance, with higher values often suggesting increased sympathetic dominance. The table allows for a quantitative comparison of HRV-B and SSP interventions, highlighting their potential impact on autonomic regulation. Studies on HRV-B consistently show improvements in HRV parameters, reinforcing its established role in enhancing autonomic function. In contrast, SSP demonstrates potential effects on vagal tone but requires further investigation to confirm its efficacy through more extensive controlled trials. HRV, heart rate variability; HRV-B, heart rate variability biofeedback; SSP, safe and sound protocol; SDNN, standard deviation of normal-to-normal intervals; LF, low frequency; HF, high frequency; RMSSD, root mean square of successive differences.

**Table III:** Mechanisms and effects of HRV-B and SSP.

Intervention	Mechanism	Physiological effects	Clinical benefits	Authors, year of publication	(Refs.)
HRV-B	Baroreflex activation, vagal stimulation	Increased HRV, reduced inflammation, improved autonomic flexibility	Cardiovascular risk reduction, stress resilience, improved emotional regulation	Lehrer *et al*, 2013	(95)
SSP	Auditory vagal stimulation, neuroplasticity modulation	Enhanced social engagement, reduced stress response	Potential therapy for PTSD, ASD, and anxiety-related disorders	Porges *et al*, 2018	(104)
HRV-B	Strengthening vagus nerve and activation of the cholinergic anti-inflammatory pathway	Reduced TNF-α, increased HRV (SDNN, total power, LF)	Reduction in systemic inflammation; potential lowering of cardiovascular and metabolic disease risk in panic disorder patients	Herhaus *et al*, 2023	(118)
SSP	Modulation of the nucleus ambiguous and dorsal vagal complex	Improved self-regulation and attentional control	Increased adaptability in social and cognitive settings	Kawai *et al*, 2023	(103)

The table summarizes the proposed physiological mechanisms and clinical effects of HRV-B and SSP. HRV-B is primarily associated with baroreflex activation, vagal nerve stimulation, and modulation of the cholinergic anti-inflammatory pathway, leading to reduced inflammation and improved autonomic function. While still an emerging intervention, SSP is believed to engage auditory-vagal pathways and promote neuroplasticity within the autonomic nervous system. The clinical benefits of both interventions, including stress resilience, improved cardiovascular health, and enhanced emotional regulation, are also highlighted. HRV, heart rate variability; HRV-B, heart rate variability biofeedback; SSP, safe and sound protocol; PTSD, post-traumatic stress disorder; ASD, autism spectrum disorder.

## Data Availability

Not applicable.
